# A Needle-Free Jet Injection System for Controlled Release and Repeated Biopharmaceutical Delivery

**DOI:** 10.3390/pharmaceutics13111770

**Published:** 2021-10-22

**Authors:** Mojiz Abbas Trimzi, Young-Bog Ham

**Affiliations:** 1 Department of Plant System & Machinery, University of Science & Technology (UST), Daejeon 34113, Korea; mat@ust.ac.kr; 2 Energy Systems Research Division, Korea Institute of Machinery & Materials (KIMM), Daejeon 34103, Korea; 3 Research & Development Division, NASEM Co., Ltd., Seoul 08826, Korea

**Keywords:** needle-free, liquid jet, injection system, compressed-air and spring-driven, controlled release, repeated injection, biopharmaceutical delivery

## Abstract

Swift vaccination is necessary as a response to disease outbreaks and pandemics; otherwise, the species under attack is at risk of a high fatality rate or even mass extinction. Statistics suggest that at least 16 billion injections are administered worldwide every year. Such a high rate of needle/syringe injection administration worldwide is alarming due to the risk of needle-stick injuries, disease spread due to cross-contamination and the reuse of needles, and the misuse of needles. In addition, there are production, handling, and disposal costs. Needle phobia is an additional issue faced by many recipients of injections with needles. In addition to a detailed literature review highlighting the need for needle-free injection systems, a compressed air-driven needle-free jet injection system with a hydro-pneumatic mechanism was designed and developed by employing an axiomatic design approach. The proposed injection system has higher flexibility, uninterrupted force generation, and provides the possibility of delivering repeated injections at different tissue depths from the dermis to the muscle (depending on the drug delivery requirements) by controlling the inlet compressed air pressure. The designed needle-free jet injector consists of two primary circuits: the pneumatic and the hydraulic circuit. The pneumatic circuit is responsible for driving, pressurizing, and repeatability. The hydraulic circuit precisely injects and contains the liquid jet, allowing us to control the volume of the liquid jet at elevated pressure by offering flexibility in the dose volume per injection. Finally, in this paper we report on the successful design and working model of an air-driven needle-free jet injector for 0.2–0.5 mL drug delivery by ex vivo experimental validation.

## 1. Introduction

History dictates how outbreaks and pandemics of contagious diseases and viruses have been fatal to humankind and cattle, with the number of deaths exceeding millions of humans and animals. To date, humanity has survived influenza, typhoid fever, viral hemorrhagic fever, smallpox bubonic plague, measles, malaria, yellow fever, diphtheria, cholera, HIV/AIDS, Ebola, dengue, hepatitis, MERS, Zika virus, and coronavirus, diseases caused by some of the most destructive pathogens of all time [1,2,3,4,5,6]. For the well-being of successive generations, the fight against diseases to stop and eliminate them in the future using antibiotics, vaccines, and pharmaceuticals or through global immunization seems to be the only viable approach. The need to save human beings and animals from fatal diseases has always existed. One major contributory factor in treating infectious diseases (among other factors) is appropriate drug delivery [7,8,9]. There are many means of drug delivery [10,11]; nevertheless, the oral intake of medicine remains the most commonly used drug delivery route to date. However, the current routes of pharmaceutical administration can be categorized into intradermal and mucosal drug administration [12]. Intradermal routes of drug delivery include liquid jet injections [13,14], ballistic powder inoculation [15], and the topical administration of vaccines across the skin. In contrast, mucosal vaccination involves mucosal membranes for ocular [16,17], oral [18,19], nasal [20,21], pulmonary [22], vaginal [23], and rectal routes of pharmaceutical delivery.

Intradermal needle syringe injections become some of the most widely used means of delivering liquid drugs into the body for healthcare procedures since the invention of hypodermic needle syringes by Sir Alexander Wood of Edinburgh in 1853 [24]. Statistics suggest that at least 16 billion prophylactic and curative injections are administered worldwide every year [25]. On average, American children receive up to 26 inoculation shots for protection against diseases such as polio, measles, smallpox, influenza, cholera, and hepatitis by the time they are 2 years of age. These figures are alarming, and the use of hypodermic needles increases the global burden of diseases attributable to the use of contaminated needles for injections administered during healthcare procedures [26].

Needle-stick injuries occur accidentally in personnel-related to health care while administering injections, after the infusion, during disposal while recapping the contaminated needles, or after disposal [27,28,29,30,31,32]. Despite the everyday use of needles and sharps for injections and taking blood samples, another limitation of needle-based injection administration is needle phobia [33,34]. This is an important issue for both adults and children that makes injection administration stressful [28,35,36,37,38,39]. In addition, the economic and human costs related to injuries, wounds, needle handling, and disposal are very high [32,40,41,42,43]. The post-injury costs are estimated to range from USD 500 to USD 3000 per injury accident, depending on the treatment provided [38,42]. Furthermore, almost half of the total number of injections administered in developing and third-world countries are unsafe, leading to cases of hepatitis, human immunodeficiency virus (HIV), and more than 20 other diseases [27,30,43,44]. The World Health Organization (WHO) has analyzed the statistics regarding needle injection costs, global data on pathogen infections, and the number of deaths from unsafe injections, and has suggested that USD 14 could be saved for every single international dollar invested in injection safety [27,41,45].

The drawbacks associated with the use of hypodermic needles for drug administration have encouraged and motivated scientists to search for alternatives to hypodermic needle delivery, including the intradermal, topical, and mucosal routes. As a result of recent research there have been advances in the laser ablation of skin, passive medication absorption, iontophoresis using electric charges for enhanced drug delivery [46], electroporation for pore formation in the stratum corneum to eliminate the skin barrier [47,48,49], sonophoretic drug delivery using ultrasonic waves, microneedles of different shapes and sizes [50], and powder and liquid jet injections [51,52,53]. This article investigates the intradermal routes of inoculation and is focused on liquid jet injections [12,13,14,54]. All the routes, as mentioned earlier, involve breaking the skin barrier (which is made up of about 30 layers of dead skin cells and is known as the stratum corneum) by either degrading it or by piercing it in various ways. The stratum corneum is the first and foremost line of defense that prevents the entry and absorption of harmful foreign materials to the epidermis and tissue. It can withstand mechanical stresses ranging between 16 and 20 MPa depending upon the mechanical properties of skin, and then finally ruptures due to excessive pressure [55,56].

Passive medication absorption is the easiest and most simplistic method of drug delivery through the skin without harming the skin; however, skin permeability is usually increased using skin abrasion or some degree of laser ablation to enhance the absorption rate of the drug, and a tape or topical ointment is used to cover the affected skin part to stop possible infection and enable the passive transfer of medication to the tissue [57]. As simple as may seem, passive medication absorption is a prolonged process when it comes to the delivery rate because it can only transfer 50 milligrams of medication to the tissue over a period of 24 h [58]. The affinity of the epidermis to absorb more medicine is sometimes enhanced using iontophoresis. The mechanism of iontophoresis involves the charging of drug molecules across the skin barrier electrically to enhance the permeability of skin for increased absorption of drug molecules. The drug delivery rate using this technique is 10 times higher than that of passive absorption, reaching 500 milligrams per day, which is still considered a very slow delivery rate [46]. A mechanism involving pore generation on the skin’s superficial layer for drug deposition using pulses of electric charge is called electroporation and is similar in some ways to iontophoresis. The pores result in transfer of the drug to the tissue through electroporation in a manner much faster than that of passive absorption. Electroporation is a recent technique that requires further research to be adopted on a large scale [59]. Besides using electric charges, skin permeability can also be enhanced by ultrasonic waves and mechanical means for improved drug transfer efficacy. The mechanism involving ultrasonic waves for improved drug transfer rates across the skin barrier is termed the sonophoretic mechanism. However, it also results in the prolonged transfer of medication. Thus, sonophoretic delivery is unable to compete with needle syringe injections in terms of dose-volume delivery rates [51].

The invasive mechanisms of intradermal drug administration are becoming popular as they offer greater medication transfer rates than non-invasive intradermal drug delivery mechanisms. The most recent example of such a mechanism is the use of microneedles (MNs) fabricated with various drugs attached to multiple patches. Transcutaneous and transdermal drug delivery is made possible by the microneedles, and they provide many possibilities for multiple drug formulations and sites for patch application. Microneedles can be categorized in many ways depending on the material used for their fabrication, the area of application of microneedles, the manufacturing strategy and mechanisms adopted for the production of MNs, and finally upon their mechanical design characteristics [60]. MNs can be fabricated using various metals, polymers, glass, or silicon depending on the required characteristics of MNs, and can be used for cosmetic, therapeutic, and diagnostic purposes. With time, their areas of application may expand as they offer novel solutions for drug delivery. The production of microneedles is a cumbersome and detailed process. However, scientists have developed various microneedles by adopting etching, injection molding, micro-machining, micro-molding, and lithographic electroforming replication techniques. In addition, microneedles are available in multiple forms, for example with solid/hollow or coated/un-coated presentations (in the case of solid microneedles), as well as dissolvable or non-dissolving types of structures [61].

The reason behind the popularity of microneedles is their capacity to enter the skin and human body efficiently and effectively without drug degradation. Another reason is their pain-free drug delivery with a microscale form factor with easy operation. Typical candidates for developing novel therapeutics are large biomolecules like peptides, polypeptides, antibodies, and proteins. Nevertheless, their application and delivery options are limited due to their large size. The skin barrier (epidermis) allows and encourages the permeability of molecules with molecular weights of less than 500 Da (where 1 Dalton (Da) = 1 g/mol). Thus, there is a need to develop microneedles using various mechanisms to deliver novel medications consisting of large molecules for painless drug delivery [62].

There are some limiting factors of microneedles that need to be resolved before the commercialization of MNs. First, they have limited use as they strongly depend on the dissolution of drug molecules by skin and tissue, which can be very time-consuming. Second, solid and coated microneedles exhibit safety issues due to their tendency to shatter inside the skin, representing a biohazard. Third, hollow-structure microneedles have weak mechanical stuffiness and strength, which may cause MNs to break before penetrating the skin barrier. Fourth, the fabrication of hollow needles is also a very complex and limiting process. Finally, dissolving microneedles address the issues present in other types of MNs by incorporating a dissolvable needle tip and support layer, which provides good mechanical strength to penetrate the skin. Their dissolving capability eliminates the risk of leaving any bio-hazardous material inside the body during the insertion process, and their fabrication process is simple as well. However, there is a lack of suitable materials for dissolvable microneedles that are acceptable to tissue. The available biomaterials show weak strength and limited stability under severe application conditions. In addition, the availability of drugs ideal for the fabrication of dissolvable microneedles is minimal, or the available materials are too costly to be used as a cheaper alternative to needle syringes [63,64,65].

Liquid jet injections can deliver the vaccine to the dermal, subcutaneous, and muscular regions depending upon vaccination requirements. Ballistic inoculation involves the delivery of vaccines as a powder to the outermost layers of the skin. It has been adopted to apply rapid local analgesia on the back side of the hand using lidocaine in a powdered form [66]. However, the availability of drugs in powdered form within the recommended dose concentrations is an area that needs further attention in order for this mechanism to be expanded and used more frequently. Alternative routes deliver vaccines through the epidermis. Inoculation through the epidermis is facilitated by several mechanisms, including DNA inoculation by hair follicles, stratum corneum exposure and vaccine absorption by tape stripping, micro-pore vaccine delivery by thermal and radio wave ablation, dermal absorption enhancement for vaccine by micro-emulsions and colloidal carriers, low-frequency ultrasound utilization for vaccine absorption, dermal electroporation for DNA vaccine delivery to the epidermis, epidermal microneedles, ballistic powder jet injectors, and liquid jet injectors [67,68,69,70,71].

Recently developed methods are lacking either in delivery volume or drug availability in the desired concentrations and forms. In addition, the alternative drug delivery methods reported in literature are laboratory-based and require further time and extensive research for adaptability. However, needle-free jet injectors have been around since 1866, when the first jet injector was invented by Galante [72,73]. In his Manual of Hypodermic Medication (1879), Bartholow of Philadelphia reported an aqua-puncture instrument for the treatment of uncontrolled neuralgia which was invented by Guerart for the simultaneous introduction of several liquid jets [74]. In the 1930s jet injectors were reconsidered and utilized in vaccine delivery applications, and since then there has been continuous development in the field of needle-free jet injectors [55,75].

Liquid jet injectors (LJIs) utilize the kinetic energy of a high-velocity medication jet with a diameter that is smaller than the outer diameter of a standard 21G needle [76]. The skin is an attractive target for immunization because it is an integral part of the immune system. The epidermis is supplemented with the Langerhans cell (LC) network, a subset of immature dendritic cells residing in the epidermis, which allows them to absorb antigen efficiently and perform immune surveillance. In addition, the Langerhans cells provide preliminary defense after the physical barrier of the stratum corneum has been penetrated. Langerhans cells are vital as they initiate specific immune responses by processing and presenting antigen fragments to naive cells in the lymphatic nodes, promoting the generation of both systemic and mucosal humoral immune responses that are fundamental for the existence of the individual [77,78,79].

During pandemics like those of influenza or COVID-19 vaccine shortages may occur due to the need for the vaccination of most people. In such times of need, reduced-dose vaccine delivery may be the only solution. Thus, the vaccine should be targeted to the skin to promote the contact of vaccine with Langerhans cells, resulting in better immune response with lower doses [80,81]. In addition, vaccines injected using liquid jet injectors usually provide better dispersion throughout a large tissue volume after injection than vaccines delivered using hypodermic needles. The better tissue dispersion allows vaccines to establish better and faster contact with Langerhans and antigen-providing cells before their degradation [13].

**Figure 1:**
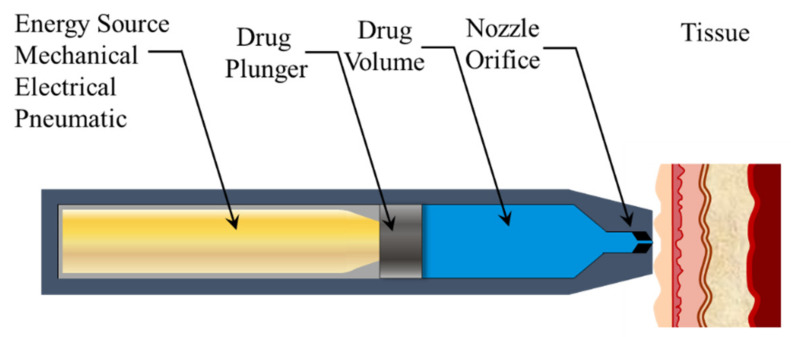
Working principle of an oversimplified model of a needle-free jet injector.

Auto-reloading jet injection systems (ARJIs) can perform 1000 injections per hour and are used in the mass vaccination of livestock. They can be used to deliver a variety of drugs like insulin, lidocaine, DNA vaccines, and anti-tumor drugs that interfere with RNA [55,82,83,84,85,86]. Typical jet injectors pressurize the liquid at around 20 MPa, and typically have a single outlet in the form of a nozzle or orifice with diameters ranging between 30 and 300 μm. The jet injectors can generate jet velocity profiles of between 100 and 350 m/s. However, jet velocities of 100 m/s are necessary to break the skin barrier, penetrate the stratum corneum, and deliver the fluid to the desired depths [55,87,88]. The basic working principle of a simplistic needle-free jet injector is elaborated in Figure 1.

A small vaccine dose in the intradermal layer can generate an equal or better immune response as compared to a large volume of vaccine injected in the subcutaneous fat layer or the muscle because of the presence of dendritic and Langerhans cells in the dermis. Variable volume delivery capability and repeatability are needed so that needle-free jet injection systems (NFJISs) can be used to expand and support the area of biomedical drug delivery through the subcutaneous and transdermal routes. Thus, this paper introduces a hybrid mechanism for NFJIS that offers variable volume injections with repeatability using compressed air and spring force combined. In addition, it provides movability for the mass immunization of herds or for carrying the injection system from one place to another. This enables the safe, easy, and quick vaccination of farm animals. The maximum dose of vaccines per shot varies between 0.2 and 0.5 mL in humans, and the same goes for young cattle, piglets, and farm animals at an early age. However, in the case of adult animals, larger volume doses are recommended. For humans, the dose range usually stays the same, with some exceptions (See Appendix A Table A1), so here a small volume-delivering needle-free injection system is designed, fabricated, and experimentally evaluated for robustness of the injection system.

The proposed NFJIS aims to provide the drug in the subcutaneous range, with some flexibility and the possibility of intramuscular injections if the injection system is operated at maximum design pressure. The NFJIS requires a relatively low pressure as the drug is aimed at shallow depths. Since the exact pressure suitable for subcutaneous injection is not fixed and varies from case to case, the inlet compressed air pressure is considered the primary factor and the determining parameter for measuring the intensification caused by the mechanism [89].

The developed NFJIS can deliver a 0.2–0.5 mL injection volume by an inlet compressed air pressure of 0.20–0.50 MPa. However, ± 0.1 mL was added as a safety factor during the design phase. Thus, the design target was a 0.1–0.6 mL drug-delivering injection system. The injection system has a piston-plunger assembly responsible for drug uptake, pressurization, and injection. The mechanism has a low-pressure piston driven by pneumatic pressure, and the high-pressure plunger is in direct contact with the drug. The rod of the low-pressure piston acts as the high-pressure plunger, and both the plunger and piston are in mechanical contact and hooked together. The cross-sectional area ratio of the low-pressure piston to the high-pressure plunger is about the same as the amplification ratio. One essential design parameter is the pressure intensification ratio, which is considered to be approximately 90 times on average [90]. Besides the design optimization and development of the NFJIS, the drug delivery capability of the developed needle-free jet injection system was validated through ex vivo experiments on porcine tissue, and the injection volume control was experimentally evaluated in detail.

## 2. Mechanism of NFJIS

**Figure 2:**
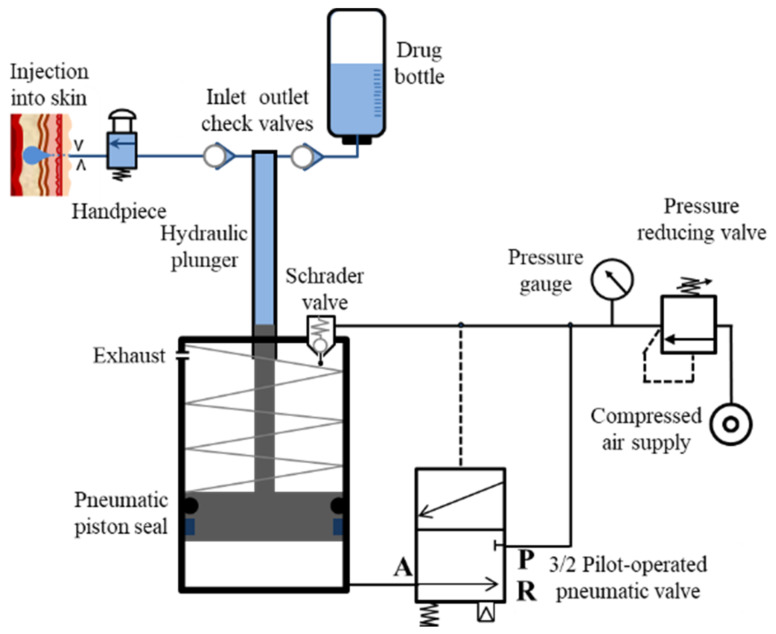
Working mechanism of the developed needle-free jet injection system.

The compressed air cylinder and pressure intensifier components include a pressure gauge and a pressure-reducing valve that extends into 2 pneumatic lines, with 1 connecting to the pressure intensifier’s Schrader valve via a small orifice and the other to the P-port of an SMC Inc. (Tokyo, Japan) 3-port 2-way pilot-operated pneumatic valve, as shown in Figure 2 (Detailed specs of valve are tabulated in Supplementary Materials).

When 0.15 MPa of pressure is accumulated in the pilot line with a short time delay (which can be modified by introducing and altering the orifice diameter in the supply line after the pressure gauge), this automated process keeps repeating consecutively at the expense of compressed air.

**Figure 3:**
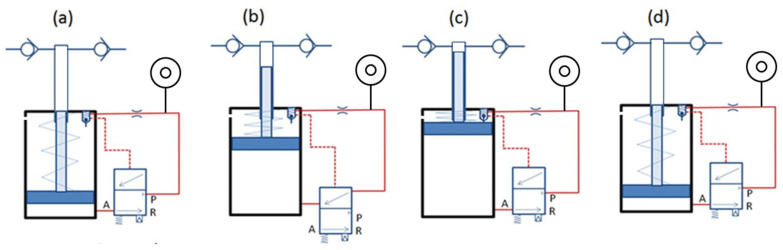
Working of the proposed needle-free jet injection system: (**a**) activation, (**b**) pressurization, (**c**) suction, (**d**) recharging.

The mechanism of auto-reloading of the needle-free jet injector is driven by compressed air [91]. Compressed air leaves the pressure-reducing valve at 0.2 MPa or more and passes through the orifice as the second line connects with port P (a). When the pressure in the pilot line increases up to 0.15 MPa, the spool of the pilot valve moves down and connects port A and port P of the valve (b). The air pushes the low-pressure piston of the pressure intensifier against the spring force until the piston touches the Schrader valve, which results in pressurization of the drug due to movement of the drug plunger (c). The opening of Schrader valves relieves the air pressure from the pilot line through the exhaust; thus, the pressure in the pilot line drops, and the low-pressure pneumatic piston moves towards the bottom due to spring force, which injects or sucks the drug through the inlet check valve in the drug chamber above the drug plunger. Hence, the gas piston returns to its initial position (d). Figure 3 depicts the entire four-step process of auto-reloading of the needle-free jet injector.

## 3. Materials and Methods for the Development of NFJIS

For the development of a needle-free jet injection system, the axiomatic design approach was adopted. Axiomatic design is a well-known design methodology that Suh first introduced in the late 1970s [92,93].

### 3.1. Axiomatic Design and Mathematical Modeling

The 3 fundamental aspects considered in the functional, physical, and process domains of the axiomatic design approach include applicable requirements, design parameters, and physical constraints. The functional requirements of the NFJIS are a 0.2–0.5 mL injection volume, 14.35–44.36 MPa output pressure, and a compact size with portability. Preliminary design parameters include the low-pressure piston-cylinder diameter (d_1_), high-pressure plunger diameter (d_2_), compressed air inlet pressure (P_1_), drug pressure (P_2_), and stroke length (S_p_). The most crucial parameter is the injection volume, defined by the stroke length and diameter of the high-pressure piston. A simplistic approach is to select a high-pressure plunger diameter and then consider the range of stroke lengths to fulfil the functional requirements of the injection volume. The inlet air pressure can be controlled so that in addition the diameter of the high-pressure plunger and the stroke range of the piston are readily selected. Furthermore, 2 crucial parameters that need to be decided include the pressure of the drug and desired intensification, and both depend entirely on the diameter of the low-pressure piston. Thus, all the parameters can be sorted out 1 by 1 using the simplistic axiomatic design model.

#### 3.1.1. Force Balance for Pressure Intensification

**Figure 4:**
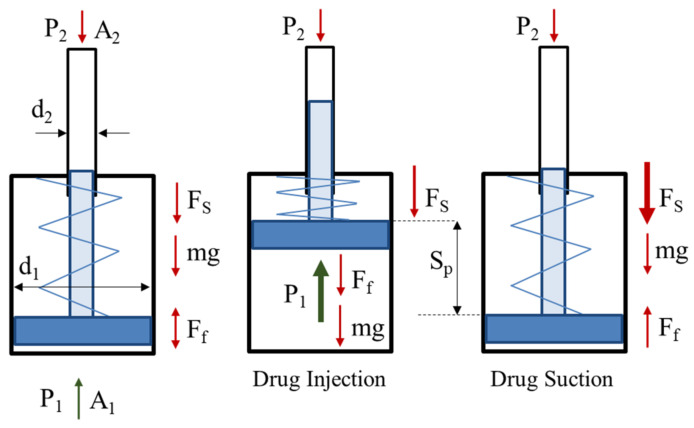
Force balance over the piston plunger of the needle-free jet injector.

The force balance over the needle-free injection system is represented in Figure 4. The governing equations for the designs of small volume delivery needle-free jet injection systems are given as follows.

(1) $$\sum F_{y}=F_{g}+F_{L}+F_{s}+F_{f}+mg$$

The equation can be rewritten as:

(2) $$\sum F_{y}=P_{1}A_{1}+P_{2}A_{2}+F_{s}+F_{f}+mg$$

As the mass of the piston and plunger is small (about 0.2 kg), the influence of gravity is minor and adds only 1.962 N force, which is a comparatively small value and can be neglected. Still, its influence is considered in output pressure calculations. The functional requirements and design parameters are selected, and the physical constraints include 0.2–0.5 MPa inlet compressed air pressure. In addition, the friction force is considered to cause a maximum of 5% of output pressure dissipation. The initial spring force is 106.5 N, with an initial compression length of 12 mm and further compression due to stroke of 38 mm with a spring constant of 2.13 N/mm.

#### 3.1.2. Design Parameters

**Figure 5:**
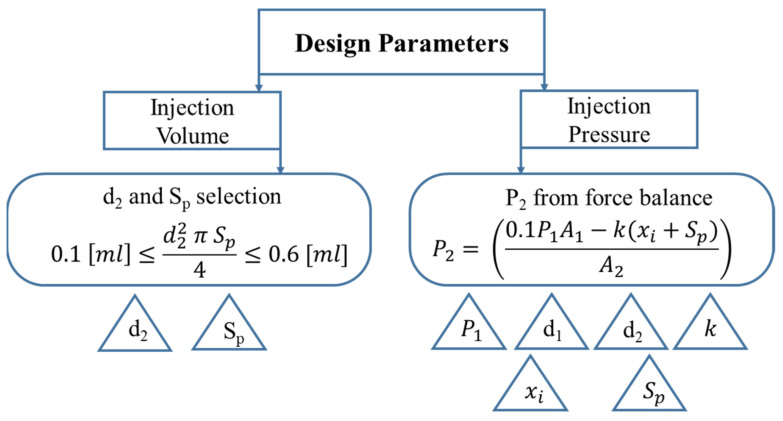
Hierarchy for NFJIS design parameter selection.

The most crucial design parameters are the injection volume and injection pressure, which are dependent on various factors as described in Figure 5.

There is one more design constraint that is not in the physical or functional domain but in the consumer domain. This is the size of the injection system.

(3) $$Intensifier\;Size\left(V\right)=A_{1}\times \left(S_{p}+\beta \right)$$

The drug delivery capacity of the needle-free injection system model is given by:

(4) $$0.1\;[ml]\leq \frac{d_{2}^{2}\pi S_{p}}{4}\leq 0.6\;[ml]\;$$

The output pressure or drug pressure expression is derived from force balance expression, considering the frictional losses.

(5) $$P_{2}=\left(\frac{0.1P_{1}A_{1}-k\left(x_{i}+S_{p}\right)}{A_{2}}\right)\frac{1}{1.05}$$

**Figure 6:**
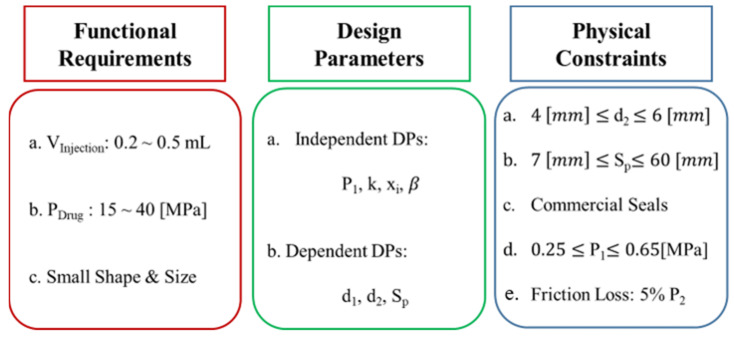
Design considerations for the needle-free jet injector using the axiomatic design approach.

The axiomatic design summary leads us towards the optimum design of NFJIS, as shown in Figure 6 below.

As the injection volume and pressure range of the small-volume NFJIS are smaller than those of its parent models, the size and shape should be more compact. It should be lighter than its large volume delivery counterparts so that handling and transportation can be more accessible. This parameter can be a functional requirement or a physical constraint, and thus dominates and leads all other factors so that the final model is compact and presentable. The overall volume of the pressure intensifier is termed as *V*, and it is defined by the cross-sectional area of the low-pressure piston times the stroke length. The factor *β* is added for the additional length of the auxiliary components and its value is found to be 183 mm. The calculation parameters are tabulated in Table 1 below.

### 3.2. Simulation Model

SimulationX software from ITI GmbH, a 1-dimensional type of commercial CFD software, was employed for system interpretation [94]. SimulationX employs a linear multistep method that uses the backward differentiation formula (BDF). Because BDF uses variable step sizes, it dramatically reduces interpretation time by increasing step sizes when the slope is not significant. Linearized and discretized expressions are analyzed using sparse matrix solvers. The ambient pressure is interpreted as the atmospheric pressure, and the temperature is fixed at a room temperature of 15 °C. The software uses the BDF up to 5 orders of magnitude, which is enough to solve interdependent complex problems. Thus, SimulationX software was used for the analysis of the designed NFJIS model [14,95,96,97].

#### 3.2.1. Pilot-Operated Pneumatic Valve

A pneumatic pilot spool operates the pneumatic valve used in the needle-free injector (NFI), and the spool returns to its normal position by the force of the spring. The core of sequence control for repeated multiple injections is the directional control valve operated by the pneumatic pilot pressure. Thus, the precise control of pilot pressure is essential. Pilot pressure is an essential factor to be considered for an appropriate time delay between the 2 consecutive injections. If the time delay is significant, there will be sufficient time for drug suction from the reservoir or drug bottle and for the piston to descend to the lowest point, but this will take long time for repeated injections. On the other hand, if the time delay is short, it is difficult for the piston descend to the bottom dead position. Even if the piston goes down, the plunger will not complete its drug suction phase, causing problems such as smaller volume injections or even no injections. The appropriate value for the time delay of this mechanism is about 0.7 s. By default, the orifice introduction to the pilot line is the most fundamental solution, as the user can adjust and use the time delay between the injections at their convenience. However, for practical use or commercialization purposes, a fixed orifice must be used to create sufficient time delay. Problems occur for a single orifice during operation, so 2 orifices and a chamber are used together to achieve a time delay of 0.7 s. Using 2 orifice diameters of 0.1–0.15 mm and 5000–10,000 mm^3^ for the chamber, a time delay of 0.7 s is achieved. The pneumatic 3/2-way control valve specifications were used by referring to the catalog of the purchased model [98].

#### 3.2.2. Pneumatic Piston

A pressure intensifier has multiple components. A hook attaches the low-pressure piston and the high-pressure plunger so when the piston rises to a certain level, the plunger also rises to that level. Their stroke is equal due to the mechanical contact between 2 components. The piston touches the Schrader valve and air escapes into the air vent. The stroke length of the low-pressure piston, the stroke length of the high-pressure plunger, and the maximum upper dead point of the Schrader valve are equal and have the same lengths. A low-pressure piston exerts a large force with low pressure and large area due to its pneumatic cross-section area. The force amplifies in the form of high pressure on the much smaller size of the high-pressure plunger. The piston rises due to the pneumatic force and returns due to the spring force, and the process is automatically repeated [89,99].

#### 3.2.3. T-Chamber

The chamber above the pressure amplifier is referred to as T-chamber, and it is generally divided into a drug suction port, 2 check valves, and dead volume. The drug suction chamber has a bottle of the drug or any liquid under experimentation mounted on it. The drug is located slightly higher than the actual supply line, so it is constantly under pressure due to its mass and height, resulting in potential energy. This pressure is caused by altitude, which prevents cavitation and helps drug suction performance by maintaining the drug flow downwards even when the system is idle.

#### 3.2.4. Hydraulic Plunger

In the hydraulic system the plunger is used in conjunction with directional control valves. Here, simple check valves are preferred over hydraulic direction control valves due to their simple structure, permitting only unidirectional flow. This helps prevent backflow and also prevents pressure leakage from the handpiece. Performance is better under slight pressure than under too-low pressure, but leakage occurs with too-high pressure. Even with the same check valve, the performance varies depending on the operating fluid. The higher the viscosity, the better the leakage prevention performance, but the losses due to pressure drop increase.

The dead volume is the cavity of the T-chamber, which can be significant when the injection volume is large, but when the injection volume is small, the dead volume also becomes small. However, if it is too small, cavitation will occur upon suction, which will cause the piston to stop at a midpoint rather than at lower point due to low volumetric elasticity when air is trapped into the T-chamber. According to the analysis, the appropriate dead volume was about 20 mm^3^, and considering the shape a value of 30 mm^3^ was expected to be reasonable.

#### 3.2.5. Handpiece

The handpiece is for repeated injection triggering. In the simulation model, an on/off valve was placed instead of a handpiece, and opened every 5 s to simulate the model’s workability. In practice, the user triggers the handpiece, and the user decides when to release the trigger.

#### 3.2.6. Simulation Model

The simulation model confirms the possibility of stable operation of the needle-free jet injection system and helps us understand the mechanism and dynamics of NFI in much better ways.

**Figure 7:**
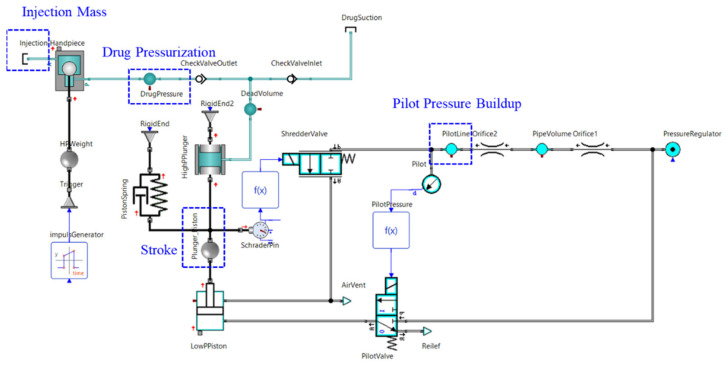
SimulationX model of NFI with data acquisition components highlighted in blue.

The simulation confirmed that repeated injections were possible once every 1.5–2.0 s. The developed model can be seen in Figure 7.

### 3.3. Vaccine and Working Fluid Properties

The variable-volume delivery needle-free jet injection system was experimentally evaluated using a vaccine named Merial 206 for foot-and-mouth disease in farm animals. The properties of the vaccine bought from domestic vendor are tabulated below in Table 2.

**Figure 8:**
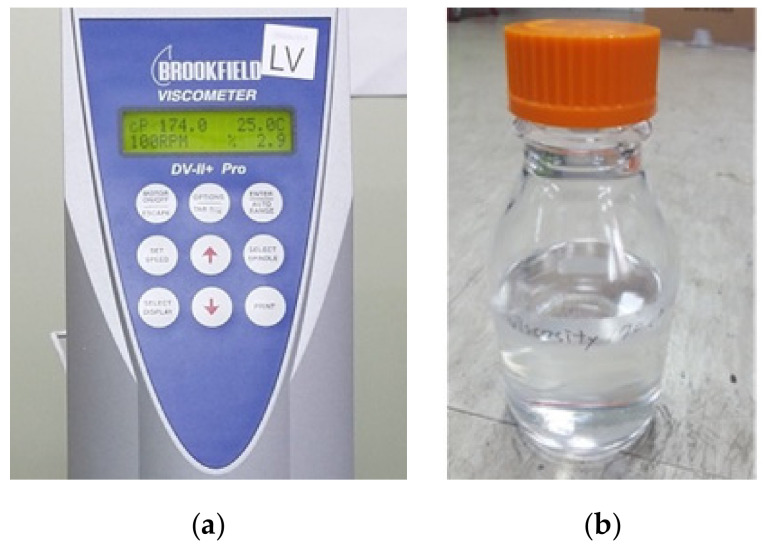
(**a**) Viscosity measurement for the vaccine, and (**b**) 170 cP liquid silicon with the same fluidic properties as the vaccine.

To avoid the wastage of vaccines during multiple calibration and performance improvement injection experiments, liquid silicon by Brookfield was used as a working fluid. Density and viscosity are essential for non-invasive injections. Density can be measured simply on a scale using a graded beaker or cylinder. However, viscosity can only be measured accurately using viscometers [100]. We used a Brookfield DV-II+ Pro viscometer for the measurement of viscosity of adopted fluid as shown in Figure 8.

First, the viscometer was calibrated by a standardized calibration process. In the calibration process, a standard-viscosity solution was added first and viscosity was measured. After adjusting the measured value to equal the standard viscosity, the viscosity was measured for the vaccine. In addition, to simulate the physical properties of the vaccine, a liquid of the same viscosity was produced using 2 Brookfield standard liquid silicones with different viscosities and then mixing them at 3000 rpm in a mixer for 60 min so that the liquid became homogeneous. 

Finally, the viscosity of the vaccine and silicon was obtained in the range of 166–175 cP. The specific gravity and viscosity values of the vaccine were 0.94 (-) and 170 (cP), and the specific gravity and viscosity values of silicon were 0.91 (-) and 170 (cP), respectively. Thus, they could be used for the injection alternatively.

## 4. Experimental Evaluation of NFJIS

### 4.1. NFJIS Troubleshooting for Normal Operation

The prototype of needle-free jet injection system was fabricated. The fabricated model was more compact than other large commercial counterparts and could be carried easily. The pressure regulator and the pressure gauge assembly used in the current model were commercial products of SMC Inc. [98].

**Figure 9:**
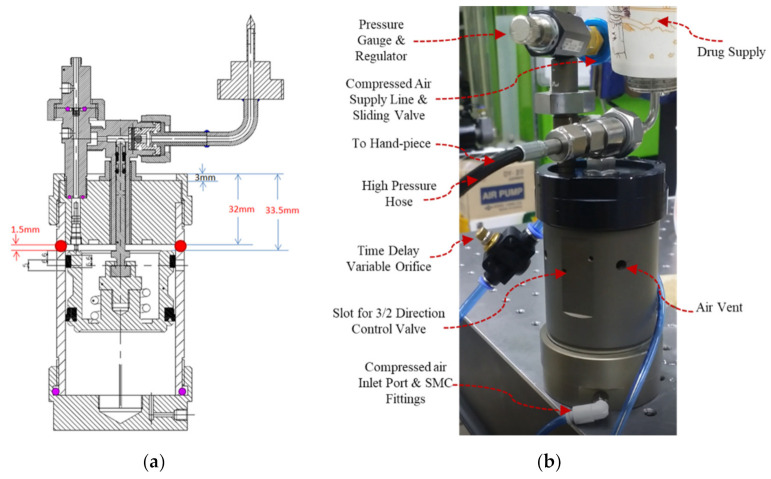
(**a**) Dimensions of a pressure intensifier for air-vent drilling in the body, and (**b**) the injection volume measurement experimental setup for 170 cP liquid silicon delivery.

During pneumatic testing it was observed that the air vent in the piston holder was blocked by the piston when it came to its top dead position. Even the Schrader valve released compressed air, but the air could not escape the amplifier. The return movement of the piston was restricted because even after the opening of the Schrader valve, the pressure in the inlet pneumatic line stayed the same, keeping the 3/2 pneumatic valve in the open position, and air from the bottom of the piston kept it in its top dead place. The problem was eliminated by drilling a new hole in the body of the pressure amplifier for the release of air coming out after the opening of Schrader valve. The purpose of the experiment was to check for an optimal place to create a new air vent to optimize the apparatus. The measurement was performed using a Vernier caliper, and the details are shown in Figure 9.

The prototype was subjected to a new air vent at a length of 32–33.5 mm below its lid. An air vent with a 1–1.25 mm diameter at a length of 32–33.5 mm was drilled. If the vent is drilled above the 32 mm length it will be occupied by the connector of the booster, and if vent is below the length of 33.5 mm, it will be closed by the guide ring of the low-pressure piston. The red circles in Figure 9a represent the conceptual position of the new air vent. After air-vent drilling the return movement problem was resolved, as shown in Figure 9b.

### 4.2. Injection Pressure and Volume Measurement

The NFJIS was prepared for injection volume measurement experiments using liquid silicon as a working fluid after troubleshooting the pneumatic circulation issue of the injection system. A close-up of the pressure intensifier with the liquid silicon supply mounted on a paper cup and the variable orifice for pneumatic circuit optimization, in addition to the high-pressure hose and compressed air supply sliding valve, can be seen in Figure 9b above. In addition to the visible apparatus, a precision balance and a sample-collecting cup lined with multiple Yuhan Kimberly wipes were used for injection volume sample collection and measurement.

A similar approach was adopted for vaccine injection experiments. Instead of the paper cup, a vaccine bottle was mounted on the drug supply housing (the handpiece can also be spotted in the Figure above). Similar to the previous experiment and the visible apparatus, a precision balance and a sample-collecting cup lined with multiple Yuhan Kimberly wipes was used for injection-volume sample collection and measurement.

The SimulationX model was used to fabricate the dose-limiters of 2 lengths, each responsible for delivering the selected dose depending on the dose-limiter attached to the low-pressure end of the pressure intensifier in the NFJIS. The experiments to check the workability of dose-limiters were carried out similarly to the injection volume measurement experiments. Each dose-limiter was plugged into the low-pressure end of the pressure amplifier to limit the injection system’s stroke. The injection was administered into a collecting cup, and injection mass was measured using precision balance for injected fluid. Finally, the injection mass measurements for each dose limiter were converted into injection volume by dividing the injection mass with the density of working fluid [85].

The injection volume measurement for each dose-limiter was carried out 20 times, and both 0.2 mL and 0.5 mL dose limiters analyzed by limiting the stroke size. All these experiments were carried out at an inlet compressed pressure of 3.0 bar, and a variable orifice was adopted for proper sequence control of the injections. The threaded grooves in each dose limiter became part of the pressure intensifier body, leaving behind the remaining length of the dose limiter for the stroke restriction. The sizes of dose limiters were selected in such a way as to accommodate excessive stroke (0.1–0.6 mL), which was left as a safety measure when designing the 0.2–0.5 mL-delivering NFI [101].

### 4.3. Ex Vivo Injection Penetration Experiments

The ex vivo experiments were performed to confirm the drug delivery capability of the developed NFJIS reported in this research. The experiments were carried out by injecting foot-and-mouth disease vaccine into MEDI KINETICS porcine skin specimens. The experimental method and conditions are listed below:

① Prepare a scale with a resolution of 0.0001 g or more and a porcine skin specimen.

② Find the density of the drug to be injected using a beaker and a scale (g/mL).

③ Set the supply pneumatic pressure for drug injection (0.25~0.35) MPa.

④ Measure the mass of the porcine skin specimen before injection (g).

⑤ Measure the mass after injecting the vaccine by NFJIS into the porcine skin (g).

⑥ Measure the tissue mass after removing the drug that could not be injected (g).

⑦ Repeat steps ④~⑥for 0.2 mL/shot and 0.5 mL/shot 10 times. 

Finally, arrange the measurement data using the formulae expressed in Table 3 below.

Through the ex vivo experiment and formulae mentioned above, the injection volume, drug transfer volume into porcine tissue, drug delivery efficiency, and drug delivery deviation were obtained. The mass of the specimen was measured on an electronic scale before and after injecting vaccine. In addition, the measurement was repeated after removing the excessive vaccine from the porcine tissue that could not be injected into the skin for drug transfer efficiency and deviation measurements.

## 5. Results and Discussion

This section includes a detailed discussion on the theoretical, simulation, experimental, and ex vivo study results and their physical significance, with an emphasis on the viability of the developed NFJIS.

### 5.1. Pressure Intensification

The design parameters were used to measure the pressure intensification caused by inlet compressed air pressure variation from 0.2 to 0.5 MPa. The pressure amplification achieved for 0.2 MPa was 17.72 MPa, which was 88.6 times the inlet pressure. The amplified pressure of the drug attainable at 0.35 MPa pressure input was 36.25 MPa, with an amplification ratio of 103.6 MPa. In addition, the drug pressure for an inlet pressure of 0.5 MPa could intensify the liquid up to 54.77 MPa.

**Figure 10:**
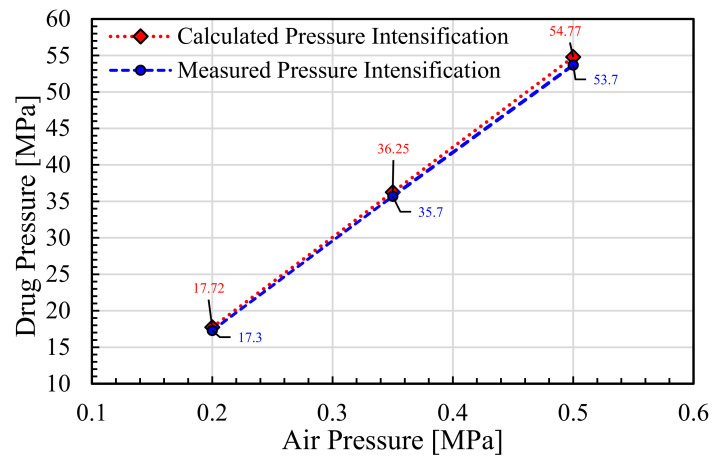
Pressure amplification results for the pressure intensifier of the NFI.

Many other factors such as exact friction losses, leakage losses, and inertial effects were ignored for ease of calculation, and the designed pressure was kept higher than the required pressure for safety reasons so that even if there were more losses than considered, the injection system would still work well. The results represent the measured and calculated pressure intensification based upon the inlet compressed air pressure, as can be seen in Figure 10.

Interestingly, the difference in measured and calculated values increased as the inlet pressure increased. The measured and calculated results at 0.2 MPa show a difference of 0.42 MPa, and the amplification achieved at 0.35 MPa inlet air pressure had an intensification difference of 0.55 MPa. In addition, the difference between measured and calculated pressure amplification reached at a maximum for an inlet air pressure of 0.5 MPa, with a value of 1.07 MPa. The gradual rise in error between the calculated values and measured values with the increase in inlet air pressure may be due to minor leakage involved in pneumatic lines. This is why the highest inlet pressure showed the biggest error with regard to the calculated and measured results. Nevertheless, a small pressure variation can be expected between numerical and experimental results when dealing with pneumatic systems at elevated pressure.

### 5.2. Simulated Model

**Figure 11:**
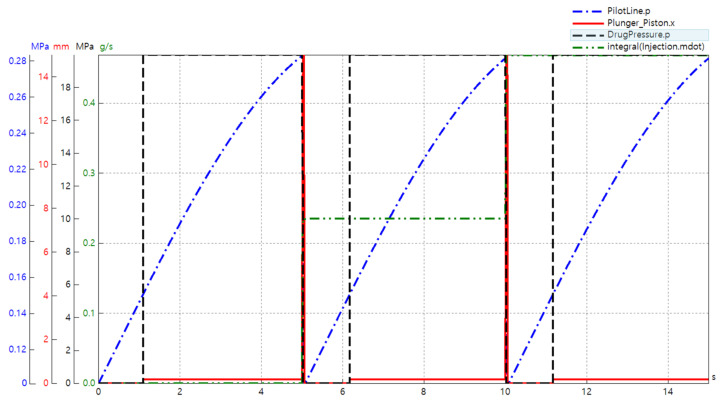
NFJIS numerical analysis results by SimulationX from the developed model.

**Figure 12:**
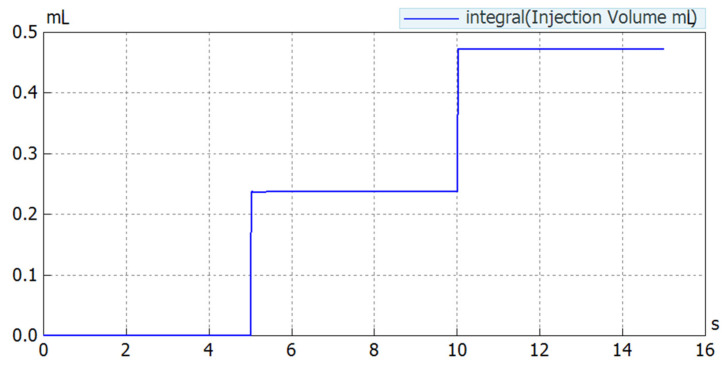
Simulation result marking flow quantity for 2 consecutive injections.

Figure 7 in the previous section represents the data acquisition components used to collect the data, and the results from the simulation described in Figure 11 below confirm the accuracy of the simulation model. The pressure in the pilot section was plotted to determine sequence control, and the reciprocating properties of the piston and plunger assembly were determined by observing the displacement of the piston. The pressure of the dead volume on the side of the handpiece could be checked to determine the pressure rise before the injection occurred, and the mass flow rate of the injectate could be integrated to calculate the total amount of the drug injected. Furthermore, the volume flow rate of the hydraulic component (denominated as drug pressure) could be integrated to solve the results for flow quantity, as shown in Figure 12 below. In addition, Figure 11 depicts the overall results of the simulation based on an inlet compressed air pressure of 0.2 MPa, which was the lowest possible pressure for the regular use of the NFJIS. Four major results are combined in one graph for the ease of verification. The graph represents the 5-second injection cycle twice altogether in one diagram, with the injection cycle repeating every 5 seconds to confirm injection repeatability.

As shown in Figure 11 above, there was no movement in the system until the pilot chamber reached a specified pressure of 0.14 MPa (the pilot pressure for the 3/2 pneumatically operated direction control valve by SMC Inc. The pilot pressure reached 0.14 MPa in about 1.9 s and pushed the spool of the directional control valve, connecting the A-port and the P-port. As a result, the air put pressure on the low-pressure piston, which rose only slightly because the plunger cavity above the high-pressure plunger had liquid present in it. This resulted in a slight increase in the volumetric elasticity or bulk modulus of the liquid. This phenomenon forced the piston to rise, causing high pressure to activate the trigger or push button of the handpiece.

After 5 seconds, the handpiece opened, relieving the pressure buildup from the handpiece line and raising the piston. The raised piston touched the Schrader valve, which reduced pilot pressure. As the pilot pressure was relieved, the piston was lowered again. At this point, the drug was taken back into the T chamber, and this operation was repeated. The whole procedure by NFJIS could be completed and repeated within 2 seconds with an inlet air pressure of as low as 0.2 MPa. As the air supply pressure rose the injection repeatability speed became faster.

### 5.3. Injection Volume Variation

Considering the volume-delivery capacity of the developed NFJIS model, a simulation was carried out to observe the possibility of controllable volume injections. Simulation results show that the delivery volume could be controlled in the 0.1–0.6 mL range due to the stroke length consideration during the modeling and design phase.

**Figure 13:**
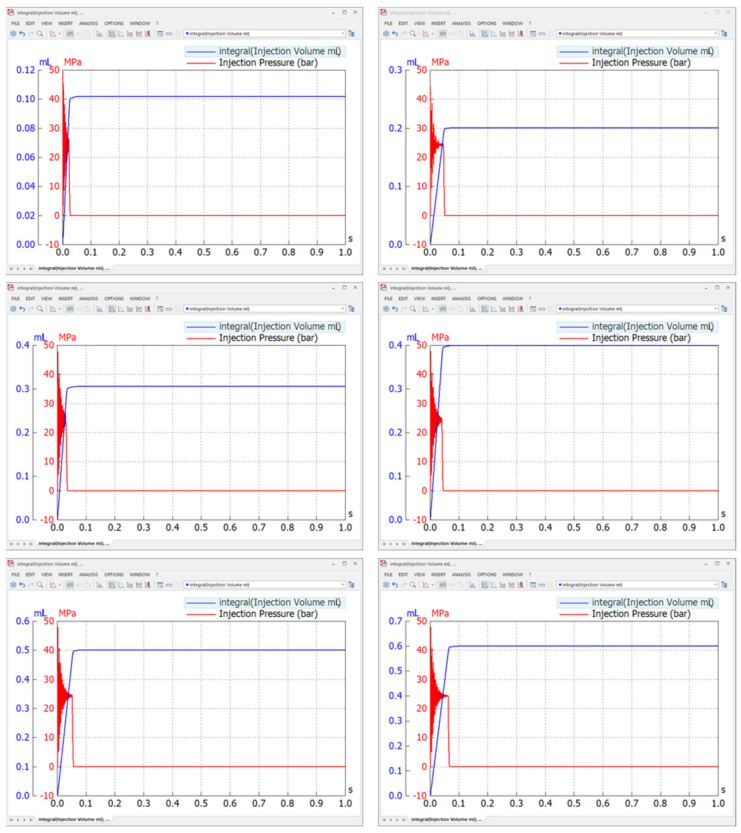
Results for injection volume control obtained through modeling of the NFI using SimulationX software.

As shown in Figure 13, the results from the simulation confirmed the possibility of volume control. However, to keep the performance evaluation simple, only two dose limiters were tested. The injection volume variation is an important and useful aspect when developing a needle-free injection system. For a variable-volume delivery needle-free jet injection system, dose limiters with threaded grooves make it possible to control the injection volume.

Experiments for variable volume drug delivery using liquid silicon and the Merial 206 vaccine were carried out. The injection volume readings for consecutive injections of each liquid up to 20 times were recorded and plotted. The average injection volume of 20 shots for liquid silicon with 170 cP viscosity with 0.2 mL capacity injections with the air-driven needle-free jet injection system was measured to be 0.2014 mL. For the injections with 0.5 mL capacity, the average injection volume was 0.4981 mL. Similarly, the results for 0.2 mL injections resulted in a maximum injection volume of 0.2133 mL and a minimum injection volume of 0.18924 mL, with another 18 values lying in between these values. However, the 0.5 mL delivery dose limiter provided maximum and minimum injection volumes of 0.5168 mL and 0.4798 mL, respectively. All these experiments were carried out at an inlet compressed air pressure of 0.3 MPa.

The Merial 206 vaccine injections were carried out with a similar experimental approach by only changing the liquid for injection volume measurement from liquid silicone to the actual vaccine. The 0.2 mL delivery injections resulted in an average injection volume of 0.2026 mL, and the 0.5 mL delivery injections resulted in an average injection volume of 0.5042 mL for 20 consecutive injections recorded at an inlet compressed air pressure of 0.3 MPa.

**Figure 14:**
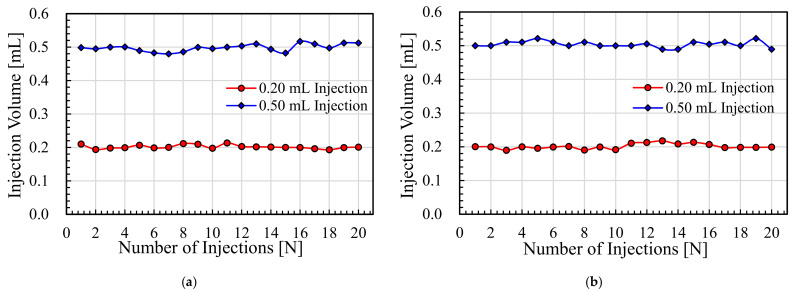
Injection volume measurement results for variable volume delivery. (**a**) Injections using 170 cP liquid silicon by Brookfield, and (**b**) injections using the Merial 206 vaccine using the NFJIS.

The maximum and minimum readings for 20 injections at 0.2 mL of injection per shot were 0.2176 mL and 0.1897 mL, respectively. Similarly, the 0.5 mL dose injections provided values of 0.5213 mL and 0.4894 mL as the maximum and minimum injection values, respectively, as shown in Figure 14 above.

### 5.4. Ex Vivo Experiments and Volume Control

The penetration capability of the needle-free jet injection system was confirmed through the ex vivo experiments. The porcine skin specimens were targeted with a series of injections at 0.2 mL/shot and 0.5 mL/shot through the developed NFJIS. The average injected vaccine volume using the NFJIS at 0.2 mL/shot was 0.1987 mL, with maximum and minimum injectate volumes of 0.227 mL and 0.182 mL, respectively. Furthermore, the average transferred vaccine volume into the porcine specimen through NFJIS for 0.2 mL/shot was 0.192 mL, with maximum and minimum delivery volumes of 0.21 mL and 0.18 mL, respectively.

**Figure 15:**
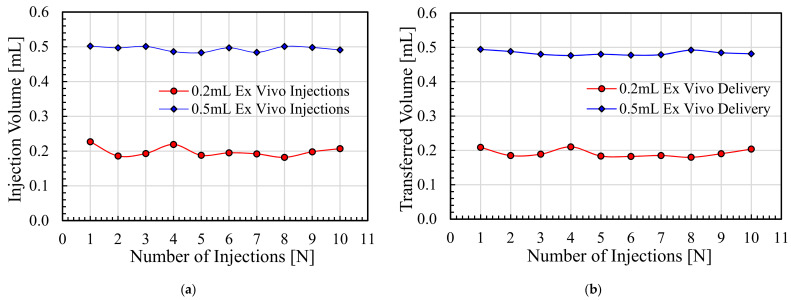
Ex vivo experiments for vaccine delivery by the NFJIS into a porcine tissue specimen. (**a**) Pharmaceutic volume ejected by NFJIS at 0.2 mL/shot and 0.5 mL/shot, and (**b**) vaccine volume transferred into the porcine tissue by the NFJIS for variable-volume shots.

Similarly, the average injected volume through NFJIS for 0.5 mL/shot was 0.494 mL, with maximum and minimum injectate volumes of 0.502 mL and 0.483 mL, respectively. However, the average transferred volume through NFJIS for 0.5 mL/shot remained at 0.483 mL, with maximum and minimum delivery values of 0.494 mL and 0.476 mL, respectively. The injected volume and transferred volume results for 10 consecutive vaccine injections into ex vivo porcine tissue are plotted in Figure 15a and Figure 15b, respectively.

**Figure 16:**
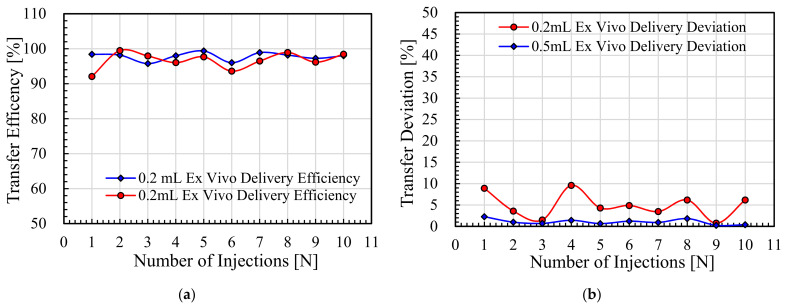
Ex vivo experimental results for Merial 206 vaccine delivery by the NFJIS into a porcine tissue specimen. (**a**) Pharmaceutic volume delivery efficiency at 0.2 mL/shot and 0.5 mL/shot, and (**b**) vaccine delivery deviation by NFJIS for variable-volume shots.

The vaccine delivery efficiency and delivery deviation values at both 0.2 mL/shot and 0.5 mL/shot for 10 consecutive shots into the porcine skin by NFJIS are shown in Figure 16a and Figure 16b, respectively.

The maximum and minimum transfer efficiency values of 10 consecutive injections at 0.2 mL injection per shot were 99.5% and 92.1%, respectively, with an average drug delivery efficiency of 96.7%. Similarly, the 0.5 mL vaccine injections provided 99.4% and 95.8% maximum and minimum drug delivery efficiency, respectively, with an average 97.8% drug transfer efficiency, as shown in Figure 16a. Similarly, the drug delivery deviation for 0.2 mL and 0.5 mL injections was experimentally evaluated with maximum values of 9.6% and 2.3%, respectively, as represented in Figure 16b.

**Figure 17:**
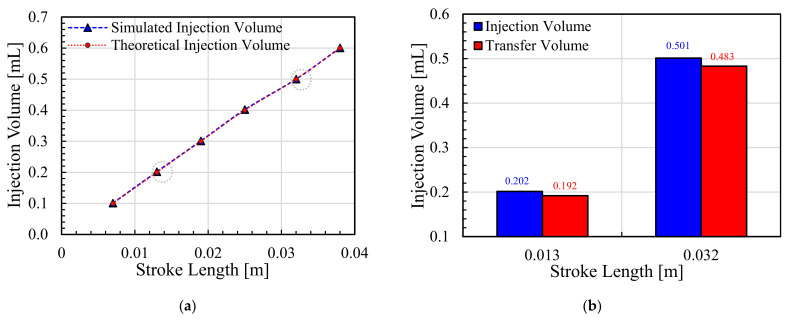
Injection volume control and variable volume delivery validation. (**a**) NFJIS volume outcome using theoretical calculations and a simulation model, and (**b**) ex vivo injected and transferred pharmaceutic volume by the NFJIS with respect to stroke length.

Interestingly, the dose-volume control range could be expanded only by fabrication of dose-limiters of variable lengths to limit the stroke of the piston-plunger for desired injection volume delivery. Figure 17a represents the calculated and simulated values for the injectable pharmaceutic volume by employing the proposed NFJIS. However, only two dose-limiters were developed to check the drug delivery capability of the NFJIS via ex vivo experiments, and average injection volumes of 0.202 mL/shot and 0.501 mL/shot were delivered by each dose-limiter due to a fixed stroke length for each case. Similarly, the drug delivery volume was minutely less than the overall injection volume, as shown in Figure 17b.

Finally, the research was validated by the theoretical calculations and simulation results, which justified the design and development approach. Similarly, the performance of the NFJIS was experimentally evaluated initially using injection pressure measurement, with pressure as the primary and most crucial parameter. In addition, the variable-volume controlled release was verified by employing 2 dose-limiters at 0.2 mL/shot and 0.5 mL/shot. Finally, the ex vivo studies confirmed the drug delivery capability and performance of the proposed needle-free jet injection system (see SM).

## 6. Conclusions

In this research, the design procedure for a needle-free jet injection system with an axiomatic design for an injection delivery volume range of 0.2–0.5 mL using an inlet compressed air pressure of 0.20–0.50 MPa is explained in detail. An extensive simulation was used for modeling and design finalization.

In addition, the model fabrication, seal selection, and troubleshooting of the injection system for better performance were addressed in detail. The simulation model helped us understand the mechanism better and successfully develop a working prototype for a 0.2–0.5 mL biopharmaceutical delivery injection system.

Furthermore, the injection depth control by inlet air pressure variation was also possible due to the current mechanism, as inlet air pressure enhances drug pressurization. This in turn is a critical factor for high-velocity jet production for penetration into greater depths. Injection volume measurement and injection volume-controlled release for repeatability and variety with regard to dose quantity were also experimentally verified for the mechanism.

The injection results showed errors of 6.3% and 7.4% for the vaccine and liquid silicon injection experiments at a 0.5 mL dosage, and 10.5% and 13.9% for liquid silicon and vaccine at a 0.2 mL dosage, respectively. The ex vivo pharmaceutic delivery results prove the capacity of the proposed NFJIS for vaccine delivery to a transdermal depth, with an average drug delivery efficiency of 96.7% for 0.2 mL/shot injections and 97.8% drug transfer efficiency at 0.5 mL/shot. The error for large-volume dose delivery seems to be less than for small-volume dose transfer, which could be improved by design optimization with careful sealing considerations in the future.

Nonetheless, the developed model could be used commercially, as the WHO guidelines for injection devices require 86% or higher accuracy, while any devices or systems below the accuracy limit are either not commercialized or are redesigned for commercialization with improved performance.

## Figures and Tables

**Table 1:** Parameters for the preliminary design of the needle-free jet injector.

Parameter	Value	Unit
HP Plunger Diameter	4.5	(mm)
LP Piston Diameter	50	(mm)
Stroke Length	38	(mm)
Inlet Pressure	0.2–0.5	(MPa)
Outlet Pressure	15–45	(MPa)
Spring Constant	2.13	(N/mm)

**Table 2:** The properties of Merial 206 vaccine for repeatability experiments with the needle-free jet injector.

Parameter	Value	Unit
Vaccine Name	Merial 206	(-)
Conductivity	0.7	(mS/cm)
Droplet Size	3956.6	(µm)
pH	8.17	(-)
Density	0.94	(g/cm^3^)
Viscosity	170	cP

**Table 3:** Equations for injection volume, transfer efficiency, and deviation measurement.

Parameter	Formula
Injection volume (mL)	(④ − ⑤) ÷ ②
Drug transfer efficiency (%)	(④ − ⑥)/(④ − ⑤) × 100
Drug transfer deviation (%)	abs.[avg.[(④ − ⑥)] − (④ − ⑥)]/avg.[(④ − ⑥)] × 100

## Data Availability

All relevant data is available within the article and Appendix A.

## Supplementary Materials

The following are available online at https://www.mdpi.com/1999-4923/13/11/1770/s1, Figure S1: The NFJIS testing for penetration possibility into porcine belly tissue; (a) porcine tissue after injections, (b) intradermal and subcutaneously injected vaccine visualization, Figure S2: The ex vivo experimental setup; (a) NFJIS ready for ex vivo experiments, (b) porcine skin sample by MEDI KINETICS, (c) porcine skin sample after 0.2 mL and 0.5 mL injections by NFJIS, Table S1: Specifications of 3-port 2-way pilot operated pneumatic valve by SMC Inc. (Tokyo, Japan), Table S2: Pressure Amplification by Pneumatic Intensifier (Reference for Figure 10 in manuscript), Table S3: Injection volume measurements results for variable volume delivery of 170 cP liquid silicon and Merial 206 vaccine (Reference for Figure 14 in the manuscript), Table S4: Ex vivo experiment results for variable volume delivery of Merial 206 vaccine (Reference for Figure 15 in the manuscript), Table S5: Ex vivo experiment results for variable volume delivery of Merial 206 vaccine (Reference for Figure 16 in the manuscript), Table S6: Comparison of results for injection volume validation and control by theoretical calculations and simulation model (Reference for Figure 17a in manuscript), Table S7: Ex-vivo injected and transferred pharmaceutic volume by NFJIS with respect to stroke length (Reference for Figure 17b in manuscript).

## Appendix A

The various diseases and their respective vaccines or curative pharmaceuticals with the dose delivery range represent the importance of the 0.2–0.5 mL volume-delivering needle-free jet injection system, as shown in Table A1.

**Table A1:** Various diseases, their drug dosage limits, and administration routes.

No.	Disease	Vaccine	Administration	Dosage
1	Chickenpox	Varicella	Subcutaneous	0.50 mL (2 doses)
2	Diphtheria	DTaP vaccine	Subcutaneous	0.50 mL (3–4 doses)
3	Tetanus	DTaP vaccine	Subcutaneous/Intramuscular	0.50 mL (4 doses)
4	Pertussis	DTaP vaccine	Subcutaneous	0.50 mL (3 doses)
5	Hib	Haemophilus influenza	Subcutaneous/Intramuscular	0.50 mL (3 doses)
6	Hepatitis A	HepA vaccine	Intramuscular	0.5–1.0 mL (2 doses)
7	Hepatitis B	HepB vaccine	Intramuscular	0.5–1.0 mL (2 doses)
8	Influenza (Flu)	Influenza vaccine	Intramuscular	0.25–0.5 mL (1 dose)
9	Measles	MMR vaccine	Subcutaneous/Intramuscular	0.50 mL (I dose)
10	Mumps	MMR II vaccine	Subcutaneous	0.50 mL (I dose)
11	Rubella	MMR II vaccine	Subcutaneous/Intramuscular	0.50 mL (I dose)
12	Pneumococcal	PCV13 vaccine	Intramuscular	0.50 mL (2 doses)
13	Rotavirus	RV vaccine	Oral	2 mL (3 doses)
14	Polio	IPV vaccine	Oral	2 drops (0.1 mL)
15	Tuberculosis	BCG vaccine	Intradermal (only)	0.05 mL–0.1 mL
16	Diabetes	Insulin	Intradermal/subcutaneous	0.01 mL–0.5 mL
