# Supplementary material for: A Needle-Free Jet Injection System for Controlled Release and Repeated Biopharmaceutical Delivery
Source: Pharmaceutics. 2021 Oct 22;13(11):1770. doi: 10.3390/pharmaceutics13111770 (PMC8620904; doi:10.3390/pharmaceutics13111770)
Supplement: Supplementary file 1 [file pharmaceutics-13-01770-s001.zip › pharmaceutics-1383789-supplementary.pdf]

pharmaceutics

MDPI

Supplementary Materials: Needle-Free Jet Injection System for Controlled Release and Repeated Bio-Pharmaceuticals Delivery

Mojiz Abbas Trimzi and Young-Bog Ham

1) Specifications of Pneumatic flow control valve:

Table S1. Specifications of 3-port 2-way pilot operated pneumatic valve by SMC Inc.

Parameter	Value
Fluid	Air
Operating pressure range (MPa)	0.15 to 0.7
Pilot pressure range (MPa)	(0.4 x P + 0.1) to 0.7, P: Operating pressure
Ambient and fluid temperature (°C)	−10 to 60 (No freezing.)
Lubrication	Not required
Mounting orientation	Unrestricted
Impact/Vibration resistance (m/s²)	300/50

2) Pressure Intensification Details:

The pressure amplification achieved for 0.2 MPa is 17.72 MPa which is 88.6 times of inlet pressure. The amplified pressure of the drug attainable at 0.35 MPa pressure input is 36.25 MPa with an amplification ratio of 103.6 MPa. In addition, the drug pressure for an inlet pressure of 0.5 MPa is capable of intensifying the liquid up to 54.77 MPa. Many other factors like exact friction losses, leakage losses, and inertial effects are ignored for ease of calculation, and the designed pressure is kept higher than required pressure for safety reasons so that even if there are more losses than considered, the injection system would still work fine. The results represent the measured and calculated pressure intensification based upon the inlet compressed air pressure as can be seen in Table S2.

Table S2. Pressure Amplification by Pneumatic Intensifier (Reference for Figure 10 in manuscript).

Inlet Air Pressure [MPa]	Calculated Pressure Boost [MPa]	Measured Pressure Boost [MPa]
0.2	17.072	17.3
0.35	36.25	35.7
0.5	54.77	53.7

It is interesting to see that the difference in measured and calculated values increases as the inlet pressure increases. The 0.2 MPa measured and calculated results show difference of 0.42 MPa, and the amplification achieved at 0.35 MPa inlet air pressure has intensification difference of 0.55 MPa. The difference between measured and calculated pressure amplification is maximum for 0.5 MPa inlet air pressure at 1.07 MPa. The gradual rise in error with the increase in inlet air pressure might be due to minor leakage involved in pneumatic lines, this is why the highest inlet pressure has biggest error between calculated and measured results. Nevertheless, small pressure variation is expected between numerical and experimental results when dealing with pneumatic systems at elevated pressures.

3) Injection Volume Variation:

The experiments for variable volume drug delivery using liquid silicon and Merial 206 vaccine were carried out. The injection volume readings for consecutive injections of each liquid up to 20 times are recorded and tabulated. The

average injection volume of 20 shots, for liquid silicon of 170 cP viscosity, for 0.2 ml capacity injections by air driven needle-free jet injection system is measured to be 0.2014 ml. For the injections of 0.5 ml capacity, the average injection volume comes out to be 0.4981ml. Similarly, the results for 0.2 ml injections resulted in a maximum injection volume of 0.2133 ml, with a minimum injection volume of 0.18924 ml, while other 18 values lie in between these values. However, the 0.5 ml delivery dose limiter provided 0.5168 ml maximum and 0.4798 ml of minimum injection volume. All these experiments were carried out at inlet compressed air pressure of 0.3 MPa.

Table S3. Injection volume measurements results for variable volume delivery of 170 cP liquid silicon and Merial 206 vaccine (Reference for Figure 14 in the manuscript).

No.	Liquid Silicone [0.2 ml]	Liquid Silicone [0.5 ml]	Vaccine [0.2 ml]	Vaccine [0.5 ml]
1	0.2102	0.4985	0.2003	0.5000
2	0.1939	0.4948	0.1996	0.5000
3	0.1981	0.4998	0.1897	0.5106
4	0.1993	0.5003	0.1998	0.5106
5	0.2067	0.4896	0.1956	0.5213
6	0.1986	0.4827	0.1992	0.5106
7	0.2003	0.4798	0.2008	0.5000
8	0.2114	0.4856	0.1983	0.5106
9	0.2097	0.4992	0.1994	0.5000
10	0.1977	0.4955	0.1914	0.5000
11	0.2133	0.4998	0.2107	0.5000
12	0.2027	0.5032	0.2128	0.5053
13	0.2018	0.5095	0.2176	0.4894
14	0.2012	0.4935	0.2084	0.4894
15	0.2000	0.4821	0.2132	0.5106
16	0.1997	0.5168	0.2069	0.5043
17	0.1963	0.5093	0.1976	0.5106
18	0.1929	0.4974	0.2139	0.5000
19	0.1924	0.5122	0.1982	0.5213
20	0.2008	0.5124	0.1989	0.4894
**Average:**	0.2014	0.4981	0.2026	0.5042
**Maximum:**	0.2133	0.5168	0.2176	0.5213
**Minimum:**	0.1924	0.4798	0.1897	0.4894
**Percentage:**	10.45%	7.40%	13.95%	6.30%

4) Ex vivo Experimental Setup and Details:

Before moving towards data curation ex vivo experiment, the developed needle-free jet injection system (NFJIS) was tested on porcine bally tissue that was purchased the same day of slaughter from the local slaughter house. The experiments were conducted on the porcine tissue and then the skin was cut using sharp blade to witness white colored vaccine presence inside the skin. The test confirmed the capability of NFJIS to pierce and penetrate the porcine skin.

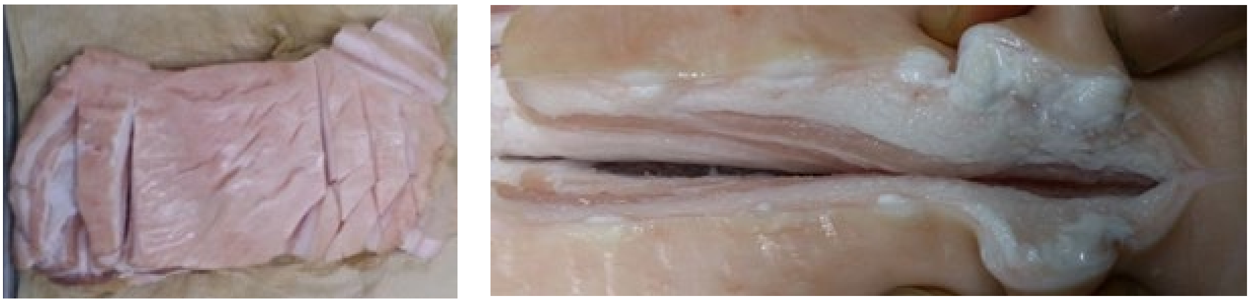

(a) (b)

Figure S1. The NFJIS testing for penetration possibility into porcine belly tissue; (a) porcine tissue after injections, (b) intradermal and subcutaneously injected vaccine visualization.

After the penetration confirmation test, the ex vivo experiments with data curation for 0.2 ml /shot and 0.5 ml/shot dose delivery efficiency, drug transfer efficiency, and drug transfer variance are conducted successfully and the results are calculated by the procedure covered in section 3.3. named 'Ex-vivo Injection Penetration Experiments'.

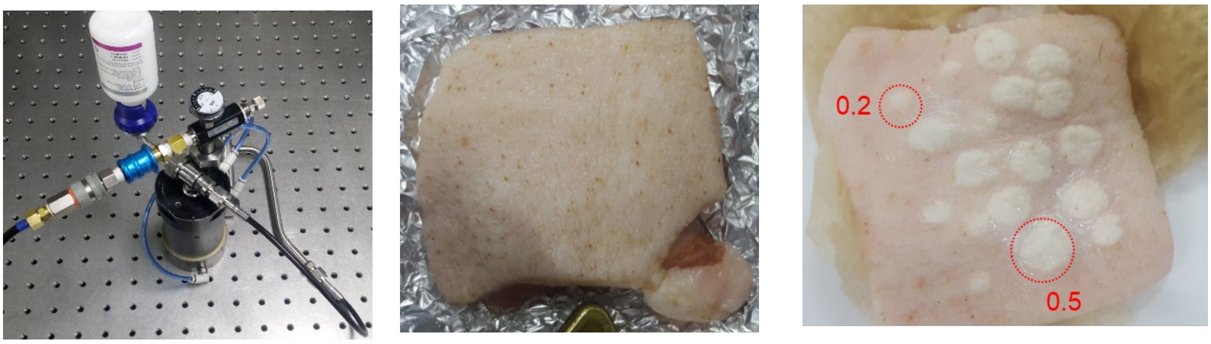

(a) (b) (c)

Figure S2. The ex vivo experimental setup; (a) NFJIS ready for *ex vivo* experiments, (b) porcine skin sample by MEDI KINETICS, (c) porcine skin sample after 0.2 ml and 0.5 ml injections by NFJIS.

Table S4. Ex vivo experiment results for variable volume delivery of Merial 206 vaccine (Reference for Figure 15 in the manuscript).

No.	0.2ml/shot	Transfer Volume [ml]	Transfer Efficiency [%]	Transfer Deviation [%]
1	0.2270	0.2090	92.0705	8.921
2	0.1860	0.1850	99.4624	3.587
3	0.1930	0.1890	97.9275	1.502
4	0.2190	0.2103	96.0274	9.598
5	0.1880	0.1836	97.6702	4.306
6	0.1950	0.1825	93.5897	4.890
7	0.1920	0.1852	96.4635	3.477
8	0.1820	0.1800	98.9121	6.182
9	0.1980	0.1904	96.1727	0.761
10	0.2070	0.2038	98.4300	6.185
**Average:**	0.1987	0.1919	96.6726	4.941
**Maximum:**	0.2270	0.2103	99.4624	9.598
**Minimum:**	0.1820	0.1800	0.4830	0.476

Table S5. Ex vivo experiment results for variable volume delivery of Merial 206 vaccine (Reference for Figure 16 in the manuscript).

No.	0.5ml/shot	Transfer Volume [ml]	Transfer Efficiency [%]	Transfer Deviation [%]

1	0.5020	0.4940	98.4064	2.259
2	0.4970	0.4879	98.1650	0.992
3	0.5010	0.4797	95.7485	0.701
4	0.4860	0.4762	97.9877	1.421
5	0.4830	0.4799	99.3582	0.660
6	0.4970	0.4772	96.0161	1.218
7	0.4840	0.4786	98.8843	0.929
8	0.5010	0.4919	98.1756	1.816
9	0.4980	0.4843	97.2490	0.251
10	0.4910	0.4812	98.0041	0.390
**Average:**	0.4940	0.4831	97.7995	1.064
**Maximum:**	0.5020	0.4940	99.3582	2.259
**Minimum:**	0.4830	0.4762	95.7485	0.251

5) Injection Volume Control:

After the penetration confirmation test, the ex vivo experiments with data curation for 0.2 ml /shot and 0.5 ml/shot dose delivery efficiency, drug transfer efficiency, and drug transfer variance are conducted successfully and the results are calculated by the procedure covered in section 3.3. named ‘Ex-vivo Injection Penetration Experiments’.

Table S6. Comparison of results for injection volume validation and control by theoretical calculations and simulation model (Reference for Figure 17a in manuscript).

Stroke [cm]	Injection Volume [ml] (Calculated)	Injection Volume [ml] (Simulated)
0.007	0.1	0.101
0.013	0.2	0.202
0.019	0.3	0.301
0.025	0.4	0.402
0.032	0.5	0.5002
0.038	0.6	0.6006

Table S7. Ex-vivo injected and transferred pharmaceutic volume by NFJIS with respect to stroke length (Reference for Figure 17b in manuscript).

Dose Limiter	Injection Volume [ml] (Experimental)	Transfer Volume [ml] (Ex vivo)
0.2 ml	0.20156	0.1919
0.5 ml	0.50115	0.4831
